# Treatment patterns in rheumatoid arthritis patients newly initiated on biologic and conventional synthetic disease-modifying antirheumatic drug therapy and enrolled in a North American clinical registry

**DOI:** 10.1186/s13075-021-02599-4

**Published:** 2021-09-08

**Authors:** Philip J. Mease, Scott Stryker, Mei Liu, Bob Salim, Sabrina Rebello, Mahdi Gharaibeh, David H. Collier

**Affiliations:** 1 Swedish Medical Center/Providence St. Joseph Health and the University of Washington, Seattle, WA 98122 USA; 2 Amgen Inc., Thousand Oaks, CA USA; 3 Corrona LLC., Waltham, MA USA; 4 Axio Research LLC, Seattle, WA USA

**Keywords:** Disease-modifying antirheumatic drug, Etanercept, Rheumatoid arthritis, US Corrona registry

## Abstract

**Background:**

Understanding the evolving treatment patterns in patients with rheumatoid arthritis (RA) is important for rheumatologists to make the best practice decisions and optimize treatment. Here, we describe treatment patterns among patients newly initiated on biologic and/or nonbiologic RA therapy over time after enrollment in the US Corrona RA registry.

**Methods:**

This was a retrospective, cohort study of adult patients with RA enrolled in the Corrona RA registry. Patients were included in this study if they initiated therapy with conventional synthetic disease-modifying antirheumatic drug (csDMARD) monotherapy, TNF inhibitor (TNFi) monotherapy, other (non-TNFi) biologic monotherapy, or combination therapy (index therapy); initiated therapy between January 1, 2004, and December 31, 2015 (index date), after enrollment in the Corrona RA registry; had at least 6 months of follow-up time after the index date; and had at least one follow-up visit. Time periods of interest were based on the year of index therapy initiation: 2004–2007, 2008–2011, and 2012–2015.

**Results:**

This study included 8027 patients. csDMARD monotherapy and TNFi + csDMARD combination therapy were the most common index therapies in the registry (39.9% and 44.9%, respectively, in the 2004–2007 period; 38.6% and 38.2%, respectively, in the 2008–2011 period; and 35.2% for both in the 2012–2015 period). At therapy initiation, a higher proportion of patients who initiated other biologics, whether as monotherapies (54.0%) or in combination with csDMARD (49.9%), had high disease activity than those who initiated csDMARD monotherapy (28.4%). For 2012–2015 vs 2004–2007 and 2008–2011 periods, persistence on a given therapy appeared to decrease for the TNFi monotherapy cohort (48.2% vs 64.3% and 52.4%) and other biologic monotherapy cohort (52.3% vs 71.4% and 54.5%) over 12 months; switching from one therapy to another was common in the Corrona RA registry.

**Conclusions:**

Increased switching from one therapy to another and decreased time on a given therapy was observed in the Corrona RA registry in the 2012–2015 period. This observation is most likely due to the increased availability of additional treatment options and/or the change in clinical focus, particularly the emphasis on achievement of treat-to-target goals of remission or low disease activity along with more aggressive treatment.

**Supplementary Information:**

The online version contains supplementary material available at 10.1186/s13075-021-02599-4.

## Introduction

Currently, multiple treatment options are available to patients with rheumatoid arthritis (RA), including conventional synthetic disease-modifying antirheumatic drugs (csDMARDs; methotrexate, leflunomide, sulfasalazine, and hydroxychloroquine), tumor necrosis factor inhibitor (TNFi) biologics (adalimumab, etanercept, infliximab, certolizumab pegol, and golimumab), and non-TNFi biologics (biologics targeting a different mechanism of action; abatacept, rituximab, anakinra, and tocilizumab) [1–3]. Targeted synthetic DMARDs (tsDMARDs) are also available, mainly the JAK inhibitors (tofacitinib, baricitinib, and upadacitinib), and a number of other JAK inhibitors are being developed for RA treatment [4–8].

While earlier clinical trials (e.g., TEMPO [9], COMET [10], and OPTIMA [11]) have reported that combination therapy of a biologic and csDMARD, usually methotrexate, is thought to be the most effective treatment option in providing remission or a low disease activity state for patients with RA, many physicians are now focusing on monotherapies, specifically biologic monotherapies, as effective options to combination therapies or csDMARD monotherapies [12, 13]. Although the American College of Rheumatology (ACR) guidelines [1] recommend csDMARDs as the first line of treatment, many rheumatologists have observed in their real-world practices that these recommendations are not always practical. This is primarily due to contraindications and the negative impact csDMARDs can have on a patient’s quality of life such as drug-induced intolerance or side effects, which then often leads to treatment discontinuation at the patient’s request [14, 15]. Furthermore, in analyses of observational registries, approximately one third of all patients with RA were already receiving biologic monotherapies, suggesting that physicians were utilizing a biologic monotherapy approach for many of their patients [12, 13].

Given the observed differences in the guideline recommendations and real-world practices, describing the evolving treatment patterns in patients with RA is essential to understand how treatment decisions are being made to optimize patient care. This study describes RA treatment patterns among patients newly initiated on biologic and/or nonbiologic therapy over time, after enrollment in the US Corrona RA registry.

## Patients and methods

### Study design and population

**Fig. 1:**
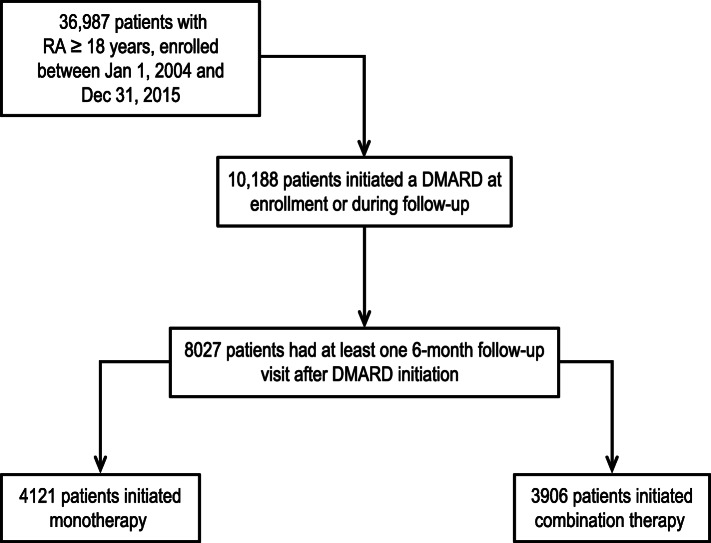
Flow of patients through the study. Patients were included in the Corrona RA registry cohort study if they initiated therapy with a csDMARD monotherapy, a biologic monotherapy, or combination therapy of a csDMARD plus a biologic between January 1, 2004, and December 31, 2015; after enrollment in the Corrona RA registry had at least 6 months of follow-up time after the index date; and had at least one follow-up visit. Patient cohorts were defined by the first treatment initiation after enrollment into Corrona and were mutually exclusive. DMARD, disease-modifying antirheumatic drug; RA, rheumatoid arthritis

This was a retrospective, cohort study of adult patients with RA enrolled in the Corrona RA registry, an independent, prospective observational cohort of patients with RA. Patients were included in this study if they initiated therapy with a csDMARD monotherapy, a biologic monotherapy, or combination therapy of a csDMARD plus a biologic (index therapy; the first therapy that the patient received); initiated therapy between January 1, 2004, and December 31, 2015 (index date; the first date of the first dose of the index therapy); after enrollment in the Corrona RA registry had at least 6 months of follow-up time after the index date; and had at least one follow-up visit (Fig. 1). For this study, combination therapy was defined as the presence of two or more agents captured during a study visit. Patient demographics and clinical characteristics were captured at registry enrollment. The Corrona questionnaires were completed by physicians and patients approximately every 6 months to capture potential therapy changes and disease activity. Of note, the Corrona RA registry did not capture data from visits that occurred outside of the Corrona visit.

Patient cohorts were defined by the first treatment initiation after enrollment into Corrona and were mutually exclusive: csDMARD monotherapy, TNFi monotherapy, other biologic (non-TNFi and tsDMARDs) monotherapy, TNFi + csDMARD combination therapy, and other biologic + csDMARD combination therapy. The TNFi + csDMARD or other biologic + csDMARD therapy groups included patients who were receiving multiple csDMARDs as part of the combination therapy. Patients could have been receiving DMARD therapy before enrollment in Corrona. Time periods of interest were based on the year of index therapy initiation: 2004–2007, 2008–2011, and 2012–2015. The patient’s index date did not have to be the same as the year of enrollment.

### Outcome measures

Patient outcomes evaluated over the time periods of 2004–2007, 2008–2011, and 2012–2015 included changes in index therapy and mean Clinical Disease Activity Index (CDAI) and therapy persistence, discontinuation, switching, and restart (defined in Table 1). Reasons for therapy discontinuation, switching, or restarting (defined in Table 1) were also evaluated.

**Table 1:** Definition of patient outcomes and reasons

Patient outcome	Definition
Persistence	Continuous use of index therapy without a treatment gap of ≥ 30 days over a 12- or 24-month follow-up period. For patients who initiated combination therapy, this could have been a treatment gap with either therapy.
Discontinuation	A gap in therapy of ≥ 30 days without another prescription for the index therapy within that period.
Switch	Initiation of a therapy—biologic or csDMARD—other than the index therapy after discontinuation of the index therapy. While patients could have more than one switch event, the outcome was defined as the proportion of patients with any switching event during the follow-up period
Restart	Discontinuing therapy for ≥ 30 days, and restarting the same therapy after the discontinuation gap
**Reasons**	
Side effect	Includes serious, minor, or fear of side effects
Social	Includes cost, preference, frequency of administration
Lack of effect	Includes inadequate response and failure to maintain initial response
Doing well	Includes remissions and similar events
Other^a^	Inclusive of all other reasons that cannot be categorized elsewhere

^a^Including therapy no longer needed, formulary restriction, patient preference, physician preference, peer suggestion, fear future side effect, patient doing well, and frequency of administration, temporary interruption, to improve compliance, to improve tolerability, route of administration

csDMARD, conventional synthetic disease-modifying antirheumatic drug

### Statistical analysis

Data were analyzed descriptively by treatment cohort. Continuous variables were described as means and standard deviations (SD). Categorical variables were expressed as percentages of the total across categories. Cumulative incidence proportions were expressed as percentages.

## Results

### Patient characteristics at index date

This study included 8027 patients (Fig. 1). Overall, mean (SD) patient age was 57 (13) years and 77% of patients were female (Table 2). There were 4541 (56.6%) patients < 60 years and 3486 (43.4%) ≥ 60 years of age. At therapy initiation, patients had mean (SD) RA disease duration of 7.9 (9.2) years, with mean (SD) CDAI of 20.0 (14.4) and Physician Global Assessment (PGA) of 34.2 (23.4). Of the 8027 patients, 4429 (55.2%) were treatment-naïve.

**Table 2:** Patient demographics and clinical characteristics at therapy initiation (2004–2015)

Characteristic	TNFi monotherapy	Other biologic monotherapy^**a**^	csDMARD monotherapy	TNFi + csDMARD combination therapy	Other biologic^**a**^ + csDMARD combination therapy	Overall
	***N*** = 770	***N*** = 369	***N*** = 2982	***N*** = 3022	***N*** = 884	***N*** = 8027
Female, *n* (%)	615 (79.9)	299 (81.0)	2264 (75.9)	2330 (77.1)	703 (79.5)	6211 (77.4)
Age (years), mean (SD)	53.6 (12.6)	58.9 (14.1)	58.1 (13.4)	56.0 (12.7)	57.8 (13.0)	56.9 (13.1)
Race, White, *n* (%)	635 (82.5)	311 (84.3)	2472 (82.9)	2460 (81.4)	731 (82.7)	6609 (82.3)
RA duration (years), mean (SD)	8.8 (9.5)	11.5 (10.1)	6.0 (8.4)	8.4 (9.1)	10.6 (9.8)	7.9 (9.2)
Number of previous therapies used, mean (SD)						
csDMARDs	1.2 (1.1)	1.5 (1.1)	0.9 (0.9)	1.5 (0.9)	1.8 (1.0)	ND
Biologics	0.8 (1.0)	1.6 (1.3)	0.2 (0.6)	0.7 (0.9)	1.6 (1.3)	ND
Previous csDMARD use, *n* (%)						
Naïve	224 (29.1)	76 (20.6)	1922 (64.5)	1748 (57.8)	459 (51.9)	4429 (55.2)
Experienced	546 (70.9)	293 (79.4)	1060 (35.5)	1274 (42.2)	425 (48.1)	3598 (44.8)
CDAI, mean (SD)	21.4 (14.7)	25.3 (14.7)	16.8 (12.9)	20.8 (14.8)	24.5 (14.5)	20.0 (14.4)
CDAI categories, *n* (%)						
Remission	72 (9.6)	15 (4.2)	310 (10.7)	224 (7.6)	28 (3.2)	649 (8.3)
Low	112 (14.9)	36 (10.0)	753 (25.9)	582 (19.7)	103 (11.8)	1586 (20.2)
Moderate	246 (32.7)	115 (31.9)	1017 (35.0)	976 (33.0)	308 (35.2)	2662 (33.9)
High	322 (42.8)	195 (54.0)	826 (28.4)	1178 (39.8)	437 (49.9)	2958 (37.7)
PGA, mean (SD)	38.1 (24.2)	42.6 (23.8)	28.8 (21.9)	35.5 (23.7)	41.2 (22.7)	34.2 (23.4)
mHAQ, mean (SD)	0.5 (0.5)	0.6 (0.6)	0.4 (0.5)	0.5 (0.5)	0.6 (0.5)	0.5 (0.5)
Comorbidities, *n* (%)						
Cardiovascular	58 (7.5)	42 (11.4)	246 (8.2)	250 (8.3)	110 (12.4)	706 (8.8)
Malignancy	30 (3.9)	35 (9.5)	228 (7.6)	150 (5.0)	68 (7.7)	511 (6.4)
Serious infection^b^	29 (3.8)	38 (10.3)	138 (4.6)	130 (4.3)	90 (10.2)	425 (5.3)
Diabetes	57 (7.4)	33 (8.9)	269 (9.0)	247 (8.2)	101 (11.4)	707 (8.8)

*N* = total number of patients in each cohort; *n* = number of patients with baseline demographic or clinical characteristic

^a^non-TNFi and tsDMARDs

^b^Not captured before 2008

*CDAI* clinical disease activity index, *csDMARD* conventional synthetic disease-modifying antirheumatic drug, *mHAQ* modified health assessment questionnaire, *ND* not determined, *PGA* physician global assessment, *RA* rheumatoid arthritis, *SD* standard deviation, *TNFi* tumor necrosis factor inhibitor, *tsDMARD* targeted synthetic disease-modifying antirheumatic drug

Within treatment cohorts, mean (SD) modified Health Assessment Questionnaire (mHAQ) score was 0.5 (0.5) for TNFi, 0.6 (0.6) for other biologics, 0.4 (0.5) for csDMARD, 0.5 (0.5) for TNFi combination, and 0.6 (0.5) for other biologic combination therapies. Additionally, for all therapy groups, approximately one third of patients were considered to have moderate disease activity based on CDAI and between 28.4% and 54.0% were considered to have high disease activity. Comorbidities among patients included cardiovascular disease (706 [8.8%]), malignancy (511 [6.4%]), serious infection (425 [5.3%]), and diabetes (707 [8.8%]). At initiation, there were fewer comorbidities in patients who started TNFi monotherapy and combination therapy than in patients who started other biologic and csDMARD monotherapies and other biologic combination therapy.

### Therapy initiation

**Fig. 2:**
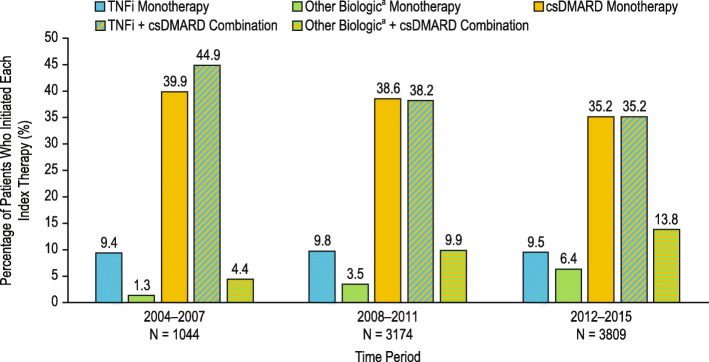
Percentage of patients who initiated each index therapy over time. The percentage of patients initiating each index therapy was determined for the treatment cohorts over the time periods of 2004–2007, 2008–2011, and 2012–2015. Patient cohorts were defined by the first treatment initiation after enrollment into Corrona and were mutually exclusive. Superscript lowercase letter “a” indicates non-TNFi and tsDMARD. csDMARD, conventional synthetic disease-modifying antirheumatic drug; *N*, number of patients in each time period; TNFi, tumor necrosis factor inhibitor; tsDMARD, targeted synthetic disease-modifying antirheumatic drug

The percentage of patients initiating each index therapy over time is shown in Fig. 2. csDMARD monotherapy and TNFi + csDMARD combination therapy were the most common index therapies in the registry, although both decreased slightly over the time periods assessed (39.9% and 44.9%, respectively, in the 2004–2007 period; 38.6% and 38.2%, respectively, in the 2008–2011 period; and 35.2% and 35.2%, respectively, in the 2012–2015 period). The percentage of patients initiating TNFi monotherapy remained stable over the time periods, while the percentage of patients initiating other biologic + csDMARD combination therapy increased. At the index date, 54.0% and 49.9% of patients were in the high CDAI category in the other biologic monotherapy and other biologic + csDMARD combination therapy cohorts, respectively, whereas PGA was similar across the cohorts (Table 2). Of the patients who were DMARD-naïve at initiation, a high proportion initiated csDMARD (64.5%) followed by csDMARD in combination with TNFi (57.8%) or other biologics (51.9%), and TNFi monotherapy (29.1%) or other biologics monotherapy (20.6%) (Table 2). Among DMARD-experienced patients, the more prior biologic therapies used, the more likely a patient was to initiate another non-TNFi biologic therapy.

### CDAI at therapy initiation over time

**Fig. 3:**
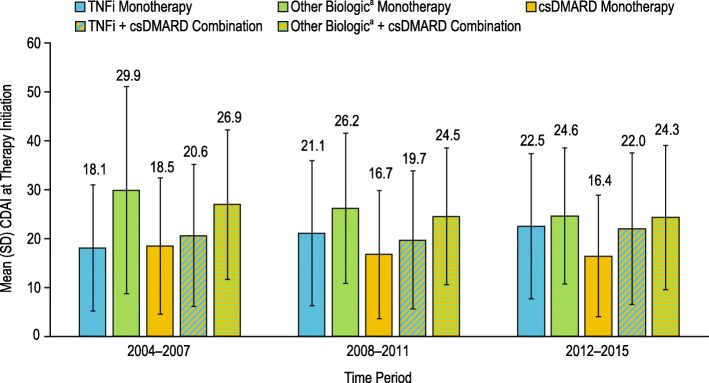
Mean CDAI at therapy initiation over time. Mean CDAI at therapy initiation was determined for the treatment cohorts over the time periods of 2004–2007, 2008–2011, and 2012–2015. Patient cohorts were defined by the first treatment initiation after enrollment into Corrona and were mutually exclusive. Superscript lowercase letter “a” indicates non-TNFi and tsDMARD. CDAI, clinical disease activity index; csDMARD, conventional synthetic disease-modifying antirheumatic drug; SD, standard deviation; TNFi, tumor necrosis factor inhibitor; tsDMARD, targeted synthetic disease-modifying antirheumatic drug

Mean CDAI at therapy initiation over time is shown in Fig. 3. Patients who initiated a biologic, either as monotherapy or combination therapy, had higher disease activity at index compared with those who initiated csDMARD monotherapy. This was observed in all three time periods. Overall, mean CDAI trended higher in patients who initiated TNFi monotherapy and lower in those who initiated csDMARD monotherapy and other biologic monotherapy.

### Persistence on therapy over time

**Fig. 4:**
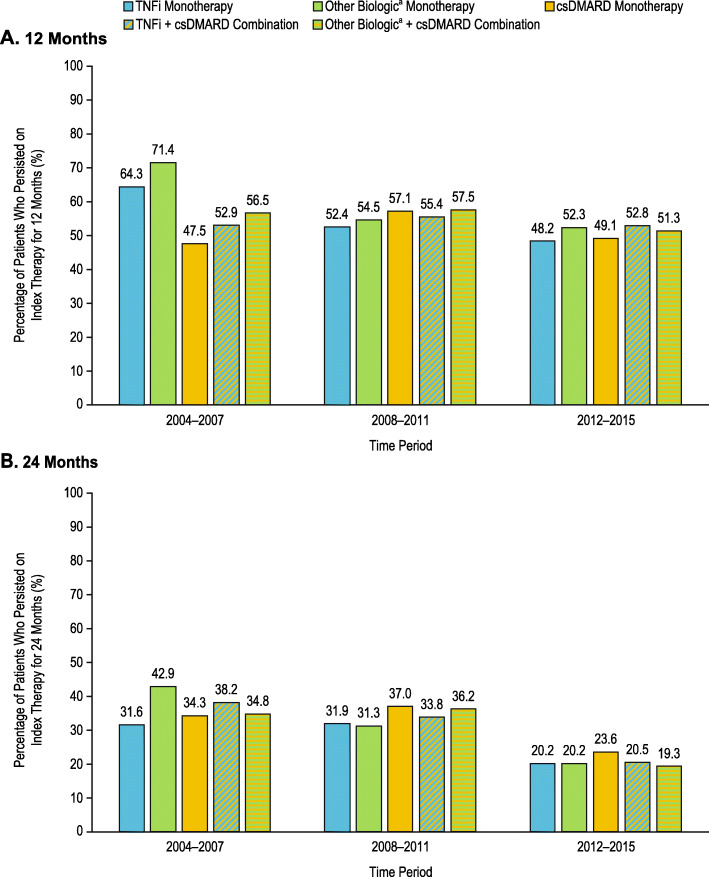
Percentage of patients who were persistent on index therapy for 12 or 24 months over time. The percentage of patients who persisted on index therapy for 12 and 24 months was determined for the treatment cohorts over the time periods of 2004–2007, 2008–2011, and 2012–2015. Patient cohorts were defined by the first treatment initiation after enrollment into Corrona and were mutually exclusive. Superscript lowercase letter “a” indicates non-TNFi and tsDMARD. csDMARD, conventional synthetic disease-modifying antirheumatic drug; TNFi, tumor necrosis factor inhibitor; tsDMARD, targeted synthetic disease-modifying antirheumatic drug

Persistence on therapy over time is shown in Fig. 4. The percentage of patients who persisted on therapy for 12 months after their index date decreased over time in the TNFi monotherapy cohort (64.3% in the 2004–2007 period; 52.4% in the 2008–2011 period; 48.2% in the 2012–2015 period) and other biologic monotherapy cohort (71.4% in the 2004–2007 period; 54.5% in the 2008–2011 period; 52.3% in the 2012–2015 period). However, this was stable or fluctuated in the csDMARD monotherapy cohort (47.5% in the 2004–2007 period; 57.1% in the 2008–2011 period; 49.1% in the 2012–2015 period), TNFi and csDMARD combination therapy cohort (52.9% in the 2004–2007 period; 57.1% in the 2008–2011 period; 49.1% in the 2012–2015 period), and other biologic and csDMARD combination therapy cohort (56.5% in the 2004–2007 period; 57.5% in the 2008–2011 period; 51.3% in the 2012–2015 period). The percentage of patients who persisted on therapy for 24 months after their index date decreased over time in all cohorts assessed, although persistence was stable through 2011 for the TNFi monotherapy and other biologic combination therapy cohorts. During the early time periods, 12-month persistence was higher in the TNFi and other biologic monotherapy cohorts than in the other cohorts.

### Discontinuation of therapy

**Fig. 5:**
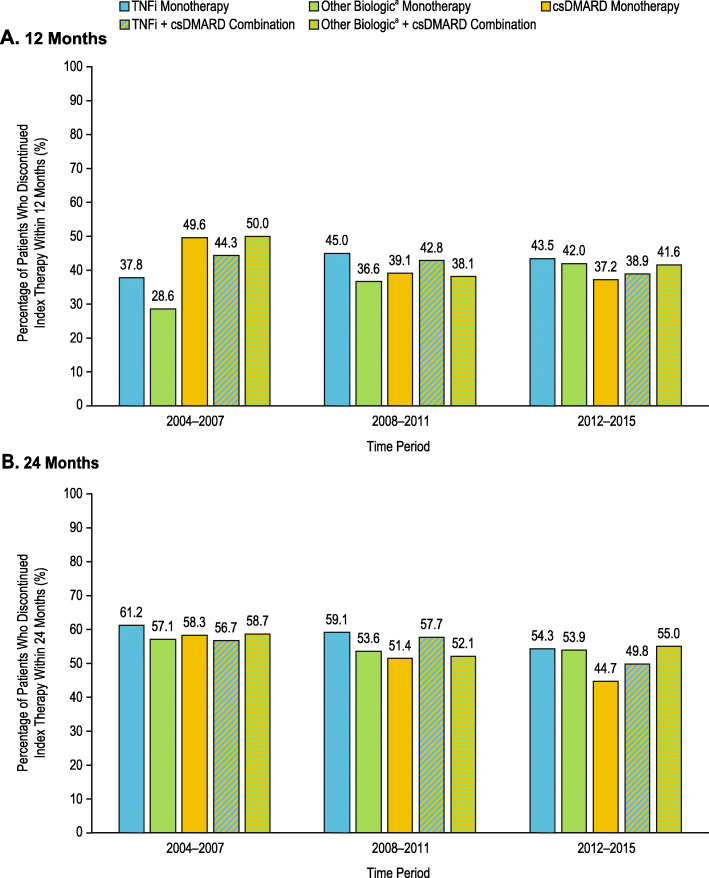
Percentage of patients who discontinued their index therapy within 12 or 24 months over time. The percentage of patients who discontinued index therapy within 12 and 24 months was determined for the treatment cohorts over the time periods of 2004–2007, 2008–2011, and 2012–2015. Patient cohorts were defined by the first treatment initiation after enrollment into Corrona and were mutually exclusive. Superscript lowercase letter “a” indicates non-TNFi and tsDMARDs. csDMARD, conventional synthetic disease-modifying antirheumatic drug; TNFi, tumor necrosis factor inhibitor; tsDMARD, targeted synthetic disease-modifying antirheumatic drug

Discontinuation of therapy within 12 or 24 months of starting index therapy is shown in Fig. 5. The percentage of patients who discontinued therapy within 12 months after their index date decreased over time in the csDMARD monotherapy (in the 2004–2007, 2008–2011, and 2012–2015 time periods was 49.6%, 39.1%, and 37.2%, respectively), appeared to be stabilized in the TNFi and csDMARD combination therapy cohort (44.3%, 42.8%, and 38.9%, respectively), and appeared to fluctuate in the other biologic and csDMARD combination therapy cohort (50.0%, 38.1%, and 41.6%, respectively). The percentage of patients who discontinued therapy within 12 months in the TNFi monotherapy cohort appeared to increase from the 2004–2007 period (37.8%) to the 2008–2011 period (45.0%), and then appeared to have stabilized by the 2012–2015 period (43.5%). The other biologic monotherapy cohort seemed to show an increasing trend over the 2004–2007, 2008–2011, and 2012–2015 time periods (28.6%, 36.6%, and 42.0%, respectively). In the csDMARD monotherapy cohort, the percentage of patients who discontinued therapy within 24 months appeared to decrease over the 2004–2007, 2008–2011, and 2012–2015 time periods (58.3%, 51.4%, and 44.7%, respectively). The percentage of patients who discontinued therapy within 24 months appeared to decrease in the TNFi monotherapy cohort over the 2004–2007, 2008–2011, and 2012–2015 time periods (61.2%, 59.1%, and 54.3%, respectively), and initially decreased for the other biologic monotherapy cohort from the 2004–2007 period to the 2008–2011 period (57.1% and 53.6%, respectively) but was stable from the 2008–2011 period to the 2012–2015 period (53.6% and 53.9%, respectively).

### Switching therapies

Among patients who initiated monotherapy, the proportion who switched to another monotherapy decreased over the course of treatment (Supplementary Figure 1). Patients who initiated TNFi or other biologic monotherapy were more likely to switch to another biologic than to a csDMARD within 12 or 24 months. Patients who initiated csDMARD monotherapy were equally likely to switch to a biologic than to another csDMARD. Patients switching from any monotherapy to csDMARD monotherapy decreased over time. Among patients who initiated combination therapy, the proportion who switched their biologic component was generally similar overtime but somewhat greater for other biologic and csDMARD combination therapy cohort in 2004–2007 (Supplementary Figure 2).

### Restarting index therapy

The proportion of patients who restarted their index therapy decreased over time regardless of the type of therapy that was initially selected (Supplementary Figure 3), except for the other biologic therapy cohort where proportions of patients restarting index therapy increased from 0% in the 2004–2007 period to 8.9% in the 2008–2011 period, and then decreased to 2.9% in the 2012–2015 period.

### Switching or discontinuing therapy

Demographics and clinical characteristics of patients who switched or discontinued therapy at the time of first switch or discontinuation are shown in Table 3. Patients who switched or discontinued their index therapy were more likely to have body mass index ≥ 25 (73.0% and 73.9%, respectively). More than a third of patients who switched (33.5%) or discontinued (36.5%) had previously received more than one TNFi; most who switched (82.7%) or discontinued (80.8%) had not received a non-TNFi biologic; and most who switched (60.5%) or discontinued (55.4%) had previously received a biologic or tsDMARD. More patients in the TNFi combination therapy cohort than the other cohorts had remission or low disease activity (LDA) at the time of switch (38.6%) or discontinuation (34.2%) (Table 3).

**Table 3:** Characteristics of patients who switched or discontinued therapy at time of first switch or discontinuation (2004–2015)

Characteristic	All switchers	Remission/LDA at time of switch	All discontinuers	Remission/LDA at time of discontinuation
	***N*** = 3520	***N*** = 1123	***N*** = 2777	***N*** = 1106
Female, *n* (%)	2798 (79.5)	866 (77.1)	2154 (77.6)	840 (75.9)
BMI, *n* (%)				
Underweight (BMI < 18.5)	48 (1.4)	11 (1.0)	39 (1.4)	11 (1.0)
Normal weight (BMI ≥ 18.5 to < 25)	901 (25.6)	322 (28.7)	685 (24.7)	296 (26.8)
Overweight (BMI ≥ 25 to < 30)	1081 (30.8)	360 (32.1)	889 (32.1)	389 (35.2)
Obese (BMI ≥ 30)	1484 (42.2)	429 (38.2)	1159 (41.8)	408 (37)
Previous number of csDMARDs, mean (SD)	0.9 (1.0)	0.9 (0.9)	0.8 (1.0)	0.8 (1.0)
Previous TNFi use, *n* (%)				
1 previous TNFi	1567 (66.5)	545 (76.2)	1079 (63.5)	480 (72.6)
2+ previous TNFi	790 (33.5)	170 (23.8)	620 (36.5)	181 (27.4)
Previous non-TNFi biologic use, *n* (%)				
0 previous non-TNFi biologic	2911 (82.7)	978 (87.1)	2243 (80.8)	950 (85.9)
1 previous non-TNFi biologic	474 (13.5)	118 (10.5)	410 (14.8)	121 (10.9)
2+ previous non-TNFi biologic	135 (3.8)	27 (2.4)	124 (4.5)	35 (3.2)
Previous biologic/tsDMARD use, *n* (%)				
1 previous biologic/tsDMARD	1510 (60.5)	537 (70.7)	1003 (55.4)	445 (64.3)
2 previous biologic/tsDMARD	560 (22.5)	140 (18.4)	453 (25.0)	154 (22.3)
3+ previous biologic/tsDMARD	424 (17.0)	83 (10.9)	353 (19.5)	93 (13.4)
Current concomitant medication, *n* (%)				
TNFi onmotherapy	399 (11.3)	121 (10.8)	253 (9.1)	105 (9.5)
TNFi combination therapy	1308 (37.2)	433 (38.6)	900 (32.4)	378 (34.2)
MTX monotherapy	363 (10.3)	125 (11.1)	390 (14.0)	135 (12.2)
MTX + other csDMARD	315 (8.9)	112 (10.0)	328 (11.8)	153 (13.8)
Other csDMARD	425 (12.1)	162 (14.4)	328 (11.8)	142 (12.8)
Disease activity, mean (SD)				
Tender joint count (28)	6.3 (7.1)	0.7 (1.2)	5.3 (6.8)	0.6 (1.1)
Swollen joint count (28)	5.1 (5.6)	0.9 (1.4)	4.4 (5.4)	0.8 (1.4)
Physician Global Assessment (0–100)	30.6 (22.6)	10.8 (10.6)	26.4 (21.8)	10.0 (10.1)
Patient Global Assessment (0–100)	42.0 (27.5)	20.9 (19.9)	38.9 (27.2)	20.9 (19.9)
CDAI	18.6 (13.9)	4.7 (3.1)	16.2 (13.3)	4.5 (3.0)
Patient pain (0–100)	44.7 (28.5)	24.5 (22.6)	41.2 (28.5)	23.5 (22.0)
DAS28	4.0 (1.6)	2.5 (1.0)	3.8 (1.6)	2.4 (1.0)
Patient reported fatigue (0–100)	47.4 (29.7)	30.4 (26.6)	44.2 (29.8)	29.1 (26.5)
hsCRP	11.8 (37.4)	11.4 (49.3)	10.9 (26.6)	9.6 (25.7)
Morning stiffness, *n* (%)	2836 (82.3)	704 (63.8)	2185 (80.6)	709 (65.5)
Morning stiffness time (hours), mean (SD)	1.6 (3.2)	0.7 (1.9)	1.6 (5.5)	0.7 (2.1)

*N* = total number of patients in each cohort; *n* = number of patients with characteristic

*BMI* body mass index, *CDAI*, Clinical Disease Activity Index, *csDMARD* conventional synthetic disease-modifying antirheumatic drug, *DAS28* Disease Activity Score-28, *hsCRP* high-sensitivity C-reactive protein, *LDA* low disease activity, *MTX* methotrexate, *SD* standard deviation, *TNFi* tumor necrosis factor inhibitor, *tsDMARD* targeted synthetic disease-modifying antirheumatic drug

### Reasons for discontinuing, switching, or adding/reducing therapy

Reasons for discontinuing, switching, or adding/reducing therapy are shown in Table 4. In the TNFi monotherapy cohort, the main reasons for discontinuation were side effects (serious, minor, or fear of side effects; 27.4%) and other reason (including therapy no longer needed, formulary restriction, patient preference, physician preference, peer suggestion, fear future side effect, patient doing well, and frequency of administration, temporary interruption, to improve compliance, to improve tolerability, route of administration; 27.4%). In the csDMARD monotherapy cohort, the main reason for discontinuation was side effects (35.7%). In the TNFi and csDMARD combination therapy cohort, the most common reasons for discontinuation were mixed (lack of efficacy, 22.2%; other reasons, 21.4%, side effects, 21.4%, and social reasons [cost, preference, or administration frequency], 27.0%). The most common reason for discontinuation was social reasons (23.9%) in other biologic monotherapy cohort and side effects (29.6%) in the other biologic and csDMARD combination therapy group. Overall, the main reasons for switching therapy were mostly due to lack of efficacy; 53.5% in the other biologic monotherapy cohort, 48.6% in the other biologic and csDMARD combination therapy cohort, 47.2% in the TNFi monotherapy cohort, 42.2% in the TNFi and csDMARD combination therapy cohort, and 28.3% in the csDMARD monotherapy cohort.

**Table 4:** Reasons for discontinuing, switching, or adding/reducing therapy (2004–2015)

	TNFi monotherapy			Other biologic monotherapy^**a**^			csDMARD monotherapy			TNFi + csDMARD combination therapy			Other Biologic^**a**^ + csDMARD combination therapy		
	***N*** = 770			***N*** = 369			***N*** = 2982			***N*** = 3022			***N*** = 884		
Factors (number of factors)	Disc. (181)	Swit. (799)	Add (383)	Disc. (97)	Swit. (258)	Add (117)	Disc. (503)	Swit. (2030)	Add (1375)	Disc. (270)	Swit. (2945)	Reduction (1567)	Disc. (68)	Swit.(762)	Reduction (438)
Number of patients with ≥ 1 reason, *n*	74	265	83	39	100	25	139	654	312	116	954	640	24	1227	165
Total number of reasons	84	390	97	46	129	32	154	821	385	126	1456	694	27	426	192
Doing well,^b^ *n* (%)	3 (3.6)	5 (1.3)	1 (1.0)	2 (4.3)	3 (2.3)	0 (0)	14 (9.1)	19 (2.3)	6 (1.6)	10 (7.9)	19 (1.3)	55 (7.9)	2 (7.4)	4 (0.9)	2 (1.0)
Lack of efficacy,^c^ *n* (%)	17 (20.2)	184 (47.2)	16 (16.5)	16 (34.8)	69 (53.5)	8 (25.0)	19 (12.3)	232 (28.3)	61 (15.8)	28 (22.2)	615 (42.2)	183 (26.4)	7 (25.9)	207 (48.6)	71 (37.0)
Other reasons,^d^ *n* (%)	23 (27.4)	96 (24.6)	61 (62.9)	7 (15.2)	20 (15.5)	15 (46.9)	34 (22.1)	196 (23.9)	236 (61.3)	27 (21.4)	360 (24.7)	186 (26.8)	5 (18.5)	89 (20.9)	42 (21.9)
Side effects,^e^ *n* (%)	23 (27.4)	61 (15.6)	9 (9.3)	10 (21.7)	18 (14.0)	3 (9.4)	55 (35.7)	250 (30.5)	28 (7.3)	27 (21.4)	266 (18.3)	142 (20.5)	8 (29.6)	80 (18.8)	40 (20.8)
Social reasons,^f^ *n* (%)	18 (21.4)	44 (11.3)	10 (10.3)	11 (23.9)	19 (14.7)	6 (18.8)	32 (20.8)	124 (15.1)	54 (14)	34 (27.0)	196 (13.5)	128 (18.4)	5 (18.5)	46 (10.8)	37 (19.3)

*N* = number of patients in each cohort; *n* = number of patients with reasons

^a^non-TNFi and tsDMARDs

^b^Doing well includes remissions and similar events

^c^Including inadequate response or failure to maintain initial response

^d^Including therapy no longer needed, formulary restriction, patient preference, physician preference, peer suggestion, fear future side effect, patient doing well, and frequency of administration, temporary interruption, to improve compliance, to improve tolerability, route of administration

^e^Serious, minor, or fear of side effects

^f^Social reasons include cost, preference, and frequency of administration

*Add* add-on, *csDMARD* conventional synthetic disease-modifying antirheumatic drug, *Disc* Discontinue, *Swit* switch, *TNFi* tumor necrosis factor inhibitor, *tsDMARD* targeted synthetic disease-modifying antirheumatic drug

## Discussion

Despite studies reporting that combination therapies are the most effective treatment regimens for patients with RA [2, 16–18], we observed that a consistent percentage of patients in the US Corrona registry started/initiated with a TNFi monotherapy; about 10% during each reported time period. Patients who initiated therapy with csDMARD monotherapy had milder disease activity than those who initiated with a biologic. Over time (2004–2007, 2008–2011, 2012–2015), treatment initiations between monotherapies and combination therapies did not change very much, except for the modest stepwise increase in combination therapy with other biologics. Initiation of combination therapy was the dominant mode of biologic therapy over the time period we analyzed, consistent with data from a number of studies [2, 16–18] and ACR guidelines that recommend use of biologics as part of combination therapies [1]. However, data coming from European and US registries and other real-world settings are increasingly showing that use of biologics as monotherapy is becoming common (with use in ~30% of patients) [19].

Switching from one combination therapy to another combination therapy was common in the Corrona RA registry. The decrease in combination therapy with TNFi and concomitant increase in combination therapy with other biologics indicates more willingness with treating RA with other biologics over time. Not surprisingly, other biologics seem to be used after csDMARDs and TNFis. This is consistent with findings from other studies that showed increased use of other biologic agents over time vs csDMARDs and TNFis [20, 21]. Once patients started on biologics, they were likely to stay on biologics rather than switch to csDMARDs. Patients starting on a csDMARD were likely to try another csDMARD or equally move on to a biologic. In general, the more severe the disease the more likely it is to use a biologic, combination biologics, or non-TNFi biologics.

Substantial differences were not observed among cohorts with regard to persistence. The proportion of patients who were persistent on their index therapy was similar between patients who initiated monotherapy and patients who initiated combination therapy in the early time periods (2004–2007 and 2008–2011). In the most recent period (2012–2015), persistence on a given biologic therapy appeared to decrease. The decrease in persistence in recent years is likely due to an increasing number of biologics and tsDMARDs becoming available for the treatment of RA and also the increased clinical focus on treat-to-target [19, 22, 23]. The current recommended treatment strategy for RA patients is to have clinicians prescribing therapies with the goal of achieving a select measurable treatment target (e.g., disease remission or LDA). Furthermore, treatment should be escalated if patients do not achieve the target [19, 22, 23], making likelihood of switching therapies more common.

Over time, patients who initiated monotherapy and then switched were more likely to go to another biologic monotherapy than to a csDMARD monotherapy. The decrease in switches to csDMARD monotherapy may have been driven by the fact that csDMARDs alone have become less acceptable as second-line therapies [24]. The introduction of tsDMARDs, mainly the JAK inhibitors for RA treatment [4–8], may have started to impact the switching patterns in the 2012–2015 time period.

Overall, patients in the TNFi combination therapy cohort had the highest remission or LDA status at the time of switch or discontinuation. There was a decreasing trend of restarting index therapy over time in the Corrona RA registry across the treatments, with 1.7–3.3% of patients restarting index therapy in the 2012–2015 period. This is several fold lower compared with the proportion of patients restarting index therapy of 13.3% reported from claims-based data for patients from 2010 to 2017 [25]. A possible explanation for the difference observed in the findings between these studies is that the Corrona RA registry did not capture data at each visit between a patient and their rheumatologist. The Corrona questionnaires were completed approximately 6 months apart. If there was a treatment gap between the Corrona questionnaire visits, it may have been captured retroactively or missed.

Treatment patterns were similar between TNFi monotherapy and combination therapies. Reasons for discontinuation were similar across studies and included lack of efficacy, side effects, and social or economic reasons such as drug cost, patient preference, and administration frequency; these are consistent with the previously published reasons for discontinuation of DMARDs [14, 22, 26, 27].

The primary strengths of this study include the large, well-defined Corrona RA registry with a systematic collection of accepted disease activity measures (i.e., CDAI). The limitations include the descriptive nature of the study and that differences are observed only, and do not take into account differences across populations, lack of visibility for patient adherence to prescribed therapy compliance and associated pharmacy records, and lack of information about changes in therapy due to changes in insurance type or formularies. Another limitation is that use of glucocorticoids at the index date was not collected and this limits the full interpretation of the data. Lastly, questionnaires were completed by physicians and patients approximately every 6 months; data from visits that occurred outside of the Corrona visit were not captured, and granular information with regard to closeness in time of two or more agents in combination therapies was not captured.

## Conclusion

In conclusion, the decrease in time on a given DMARD therapy was most likely due to the increased availability of additional treatment options and/or the change in clinical focus, particularly the emphasis on achievement of treat-to-target goals along with more aggressive treatment practices.

## Supplementary Information

**Additional file 1: Supplementary Figure 1**. Percentage of patients who initiated monotherapy and switched index therapy within 12 or 24 months over time.

**Additional file 2: Supplementary Figure 2**. Percentage of patients who initiated combination therapy and switched their index biologic within 12 or 24 months over time.

**Additional file 3: Supplementary Figure 3**. Percentage of patients who restarted index therapy within 12 or 24 months over time.

## Data Availability

The Corrona dataset is based on a large US multicenter study adhering to a number of institutional review boards, with complex logistics. Patients did not provide consent to raw data sharing during the data collection for this purpose, and the Corrona data sharing policies do not permit raw data sharing for this purpose. An aggregated limited dataset from the current analyses is available to qualified investigators with an approved protocol. Data requests may be sent to Corrona, represented by Dr. Jeffrey D. Greenberg MD MPH, NYU School of Medicine, New York, NY. E-mail: jgreenberg@corrona.org.

## Abbreviations

ACR: American College of Rheumatology
BMI: Body mass index
CDAI: Clinical Disease Activity Index
csDMARD: Conventional synthetic disease-modifying antirheumatic drug
LDA: Low disease activity
PGA: Physician Global Assessment
RA: Rheumatoid arthritis
SD: Standard deviation
TNFi: Tumor necrosis factor inhibitor
tsDMARD: Targeted synthetic disease-modifying antirheumatic drug
