# Supplementary material for: Treatment patterns in rheumatoid arthritis patients newly initiated on biologic and conventional synthetic disease-modifying antirheumatic drug therapy and enrolled in a North American clinical registry
Source: Arthritis Res Ther. 2021 Sep 8;23:236. doi: 10.1186/s13075-021-02599-4 (PMC8424897; doi:10.1186/s13075-021-02599-4)
Supplement: Supplementary file 3 [file 13075_2021_2599_MOESM3_ESM.docx]

Supplementary Figure 3. Percentage of Patients Who Restarted Index Therapy Within 12 or 24 Months Over Time

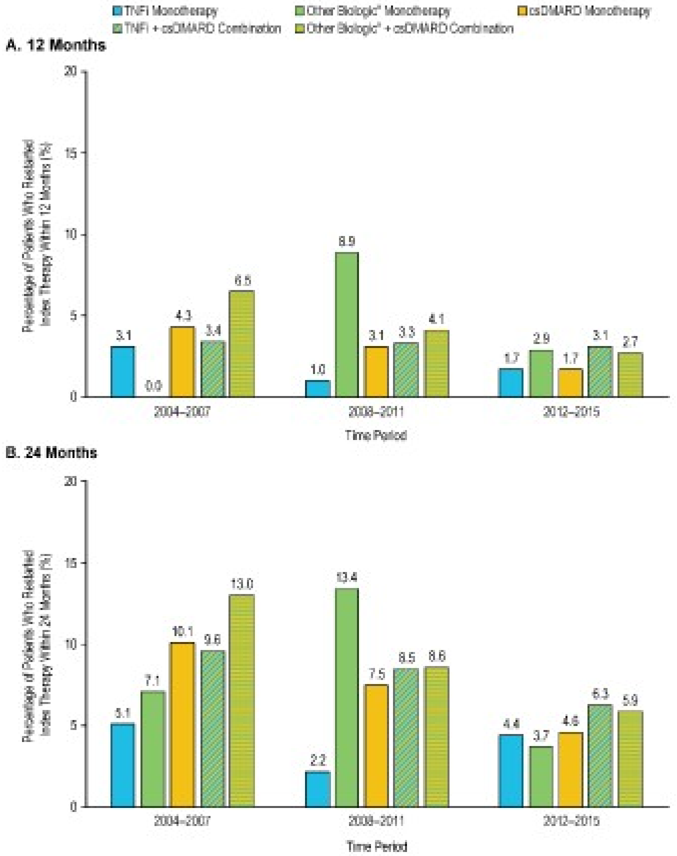

The percentage of patients who restarted index therapy within 12 and 24 months was determined for the treatment cohorts over the time periods of 2004–2007, 2008–2011, and 2012–2015. Patient cohorts were defined by the first treatment initiation after enrollment into Corrona and were mutually exclusive.

[a]Non-TNFi and tsDMARD.

csDMARD: conventional synthetic disease-modifying antirheumatic drug; TNFi: tumor necrosis factor inhibitor; tsDMARD: targeted synthetic disease-modifying antirheumatic drug.
